# Apoptotic modulators enhance oncolytic virus-induced cytokine killing in acute myeloid leukaemia (AML)

**DOI:** 10.1038/s41416-026-03417-x

**Published:** 2026-04-10

**Authors:** Basem Askar, Samuel Heaton, Tyler Barr, Richard Baugh, Noura Alzamel, Stewart McConnell, Victoria A. Jennings, Milene Volpato, Christy Ralph, Manish Jain, Richard J. Kelly, Christopher Parrish, Fiona Errington-Mais

**Affiliations:** 1 Leeds Institute of Medical Research, University of Leeds, Wellcome Trust Brenner Building, St James’s University Hospital, Leeds, UK; 2 Medical Laboratory Department, Faculty of Medical Technology, Nalut University, Nalut, Libya; 3 Department of Haematology, St. James’s University Hospital, Leeds, UK; 4 Present Address: Jack Birch Unit of Molecular Carcinogenesis, Department of Biology, University of York, York, UK

**Keywords:** Cancer immunotherapy, Cytokines

## Abstract

**Background:**

Approximately 3000 adult patients are diagnosed with AML in the UK each year. Current intensive treatments are not well-tolerated by elderly patients, and the 5-year survival rate is only 5–15%, highlighting the need for novel and effective therapies. Oncolytic viruses (OVs) preferentially replicate in cancer cells, resulting in direct oncolysis and induction of innate and adaptive anti-tumour immunity. Unfortunately, the efficacy of OVs remains relatively unexplored in AML.

**Methods:**

Using human AML cell lines, healthy-donor peripheral blood mononuclear cells (PBMC) and AML patient samples, we investigated whether combination with clinically applicable apoptotic modulators (SMAC/BH3 mimetics) can potentiate OV-induced cytokine-mediated killing.

**Results:**

We confirmed that OVs stimulate PBMCs to produce inflammatory cytokines, which can induce AML cell death. Bystander cytokine-mediated killing was also significantly enhanced in combination with SMAC/BH3 mimetics, with the optimal combination partner varying with AML subtype. We identified interferon (IFN)-α and tumour necrosis factor (TNF)-α as potential mediators of AML cytotoxicity, and SMAC/BH3 mimetics enhanced AML cell death following direct OV infection, indicating autocrine-paracrine signalling events. Pivotally, we confirmed that apoptotic modulators were effective in combination with both Live- and UV-inactivated virus.

**Conclusion:**

This work has identified a novel reovirus-based combination-immunotherapy for the treatment of AML.

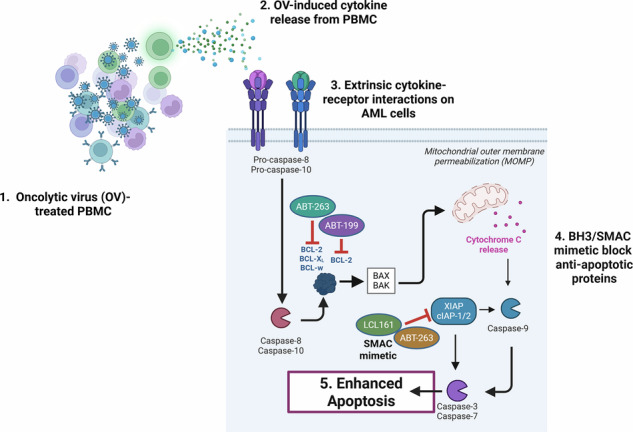

## Introduction

Acute myeloid leukaemia (AML) is characterised by uncontrolled proliferation of neoplastic myeloid cells, which accumulate in the blood and bone marrow (BM), causing anaemia, recurrent infections, bleeding and BM failure [1]. AML is the most common acute leukaemia in adults, with ~3000 new cases diagnosed in the UK each year. The most significant risk factor is age [2], although environmental factors (smoking or chemical exposure) can contribute to AML development [3]. Intensive chemotherapy regimens result in a median survival of only 8.5 months in patients aged 60 and over [4]; however, whilst newer approaches, including venetoclax/azacytidine, have improved outcomes for frail/unfit patients (median survival of ~13.6 months), they remain non-curative [5]. Therefore, AML remains a significant clinical challenge in need of novel and effective therapies [6, 7].

Chemotherapy remains the standard of care for AML and includes induction chemotherapy (cytarabine/daunorubicin) and consolidation treatment (cytarabine); however, this is not always suitable for elderly/frail patients [8]. Prognosis is linked to disease genetics, with *NRAS* and/or *RAD21* mutations associated with a favourable prognosis [9] and *IDH1/2*, *DNMT3A* or *FLT3-ITD* mutations considered high risk [10]. Consequently, targeted small molecule inhibitors have emerged as novel therapies [11] with midostaurin (a FLT3 inhibitor) [12] and ivosidenib/enasidenib (IDH1/2 inhibitors) approved for treatment [13].

Apoptotic modulators, which target BCL-2 family members and/or Inhibitors of Apoptosis Proteins [IAPs], have also been investigated in AML. The BCL-2 targeting BH3 mimetic, venetoclax (formally known as ABT-199), was recently approved in combination with azacytidine [14]. Moreover, IAPs (e.g. cellular IAP1/2 [cIAP1/2] and X-chromosome-linked IAP) are overexpressed in AML, have been associated with disease severity [15], and IAP-targeting second mitochondria-derived activator of caspases (SMAC) mimetics (BV-6/LCL161/birinapant) can sensitise AML to chemotherapy [16–19]. Importantly, SMAC mimetics have also been safe in clinical trials for other cancer types [20, 21].

Immunotherapies are also showing promise in AML [22, 23], although the efficacy of oncolytic viruses (OVs) remains relatively unexplored. OVs preferentially replicate in cancer cells, causing cell death, and induce innate (NK cell-mediated) and adaptive (T cell-mediated) anti-tumour immunity [24, 25] through the release of pro-inflammatory cytokines, tumour-associated antigens (TAA) and damage/pathogen-associated molecular patterns [24–28]. OVs have been safe and well-tolerated in clinical trials, with four OVs approved for clinical use [29]. A handful of studies also support the development of OV for AML treatment. For example: (i) myxoma virus (MYXV) prevents myeloid sarcoma, BM engraftment and reduces leukaemic burden in xenograft mice [30, 31]; (ii) coxsackievirus A21 (CVA21) induces bystander cytokine-mediated killing of AML, activates NK cell-mediated killing and primes AML-specific T cells [27]; (iii) reovirus induces direct oncolysis and inflammatory cytokine production in AML and enhances NK cell killing [32]; and (iv) UV-inactivated herpes simplex virus (HSV)-1 activates NK cells causing leukaemic cell death [33]. Enhanced immune killing has also been reported for vesicular stomatitis virus (VSV)-IFNβ-NIS when used in combination with anti-PD-L1 in a syngeneic AML model [34].

To date, the efficacy of OV (VSVΔM51/alphavirus M1) in combination with SMAC mimetics has yielded promising results in a range of cancers (breast, glioblastoma and colorectal) [35–37], and limited studies have also demonstrated that BH3 mimetics can sensitise cells to OV-mediated lysis [38–40]. Therefore, given the urgent need for novel and effective AML therapies, this study aimed to investigate the efficacy of OVs in combination with clinically applicable BH3/SMAC mimetics for the treatment of AML.

## Methods

### Cell culture

THP-1 (M5, acute monocytic leukaemia), KG-1 (M6, acute erythroid leukaemia), HL-60 and Kasumi-1 (M2, AML with differentiation) were maintained in Roswell Park Memorial Institute (RPMI)-1640 (Sigma-Aldrich) media supplemented with 10% heat-inactivated (30 min/56 °C) foetal calf serum (FCS, Gibco). Cells were obtained from the Cancer Research UK cell bank, and genotype validation was performed using short tandem repeat (STR) profiling. Mycoplasma testing was performed, and all cell lines were free from contamination. AML cell lines were seeded at 1 × 10^6^ cells/mL, unless otherwise stated.

Healthy-donor (HD) blood was obtained from National Health Service Blood and Transplant apheresis cones. PBMC were isolated using Lymphoprep^TM^ (STEMCELL Technologies) density gradient centrifugation and cultured at 2 × 10^6^ cells/mL in RPMI-1640 supplemented with 10% FCS. PBMC containing neoplastic myeloid cells were collected from AML patients diagnosed at St. James’s University Hospital using Lymphoprep^TM^. Cells were resuspended in RPMI-1640 supplemented with 20% FCS and used immediately; patient PBMCs were used at 2 × 10^6^ cells/mL. Written informed consent was obtained from all patients in accordance with institutional ethics review and approval (Leeds Teaching Hospital NHS Trust [LTHT], Research and Innovation [R&I]; LTHT R&I number, CO06/7573 and Research Ethics Committee (REC) number, 06/Q1206/106).

### Oncolytic viruses

CVA21 (*Kuykendall* strain) was purchased from ATCC and propagated/titred in-house using Mel624 cells. Reovirus type 3 Dearing and HSV-1 (HSV1716) were kindly provided by Oncolytics Biotech Inc and Biovex, respectively.

### Cell treatments

PBMC-conditioned media (PBMC-CM) were generated using HD PBMC treated with 0.1 or 1 pfu/cell OV for 48 h; cell-free supernatant was harvested and stored at −20 °C. To inactivate OV, PBMC-CM (and stock reovirus, when required) was UV-irradiated for 2 min using a C-1000 UV CrossLinker (UVP). Supplementary Fig. 1 shows that UV-irradiation of Reovirus- and HSV-1-treated PBMC-CM prevents successful viral replication, as expected [28, 41]. UV-irradiation of CVA21-treated PBMC-CM for 2 min did not prevent CVA21 viral replication (data not shown). However, AML cell lines are resistant to CVA21-mediated oncolysis, due to low/absent ICAM-1 expression (the CVA21 viral entry receptor) [27]; therefore, UV-inactivation was unnecessary to prevent replication-dependent cell death. SMAC (LCL161/BV-6) and BH3 mimetics (ABT-199/ABT-263) were obtained from Selleckchem (10 mM in dimethyl sulfoxide [DMSO]). Recombinant human cytokines (IFN-α/IFN-γ/TNF-α, R&D Systems) were reconstituted in phosphate-buffered saline (PBS) containing 0.1% bovine serum albumin; cells were treated with 500 pg/mL IFN-α/TNF-α or 250 pg/mL IFN-γ.

### Flow cytometry analysis

All flow cytometry was performed using a 6-laser Cytoflex LX (Beckman Coulter) with data analysis performed using CytExpert software.

#### Cell viability staining

Cell viability was determined using the LIVE/DEAD® Fixable Yellow Dead Cell Stain Kit (Invitrogen) according to the manufacturer’s instructions. All cells were fixed in 1% paraformaldehyde (PFA) prior to acquisition.

#### Cell phenotyping

Cells were washed in FACS buffer (PBS, 1% FCS, 0.1% sodium azide) and labelled with fluorescently conjugated antibodies (Supplementary Table 1). CD69 expression was measured on CD3^-^/CD56^+^ NK cells, alongside an isotype control. All cells were labelled for 30 min at 4 °C, washed with FACS buffer and fixed in 1% PFA.

### Cytokine detection

#### Enzyme-linked immunosorbent assay (ELISA)

Nunc Maxisorp plates were coated with capture antibodies and incubated overnight. Antibody-coated plates were blocked with 10% FCS (in PBS) and incubated with recombinant protein standards (halving serial dilutions) or sample supernatants overnight. Plates were subsequently incubated with biotinylated detection antibodies (2 h), followed by extravidin-alkaline phosphatase (ALP) conjugate (Sigma-Aldrich; diluted 1:5000 with PBS-T) for 1 h. Finally, substrate solution (1 mg/mL p-nitrophenyl phosphate (Sigma-Aldrich)) was added, and optical density readings were measured using a Multiskan EX plate reader (ThermoFisher) at 405 nm. See supplementary Tables 2 and 3 for details of matched-paired ELISA antibodies and recombinant standards, respectively.

#### Biorad multiplex assay

Production of IFN-α2, CXCL10 and TRAIL was determined using a Biorad multiplex assay (Bio-Rad Laboratories) according to the manufacturer’s instructions.

### Statistical analysis

Sample sizes were chosen based on the reproducible nature of working with established cell line models and HD PBMCs. Preliminary findings with AML patient samples are shown; however, given the highly heterogeneous nature of AML, future studies will use this data to perform power calculations and establish sample sizes to increase the statistical power of this aspect of the study. AML patient samples were excluded if cell viability without treatment was less than 50%. *P* values were obtained using one- or two-way analysis of variance (ANOVA), assuming equal variability between groups, and post-hoc testing was performed to adjust for multiple comparisons. All statistical tests were performed using Graph Pad Prism (version 10.1.0), and statistical significance is indicated when *p* ≥ 0.05. Figure legends state the statistical test used and include details of the post-hoc test used. Statistically significant results are illustrated on each figure, denoted as *p ≤ 0.05, **p ≤ 0.01, ***p ≤ 0.001 and ****p ≤ 0.0001. Figure legends also state the number of biological repeats performed for each experiment, with figures displaying individual data points for each biological repeat; bars/lines indicate mean values, ± the standard error of the mean (SEM).

## Results

**Fig. 1:**
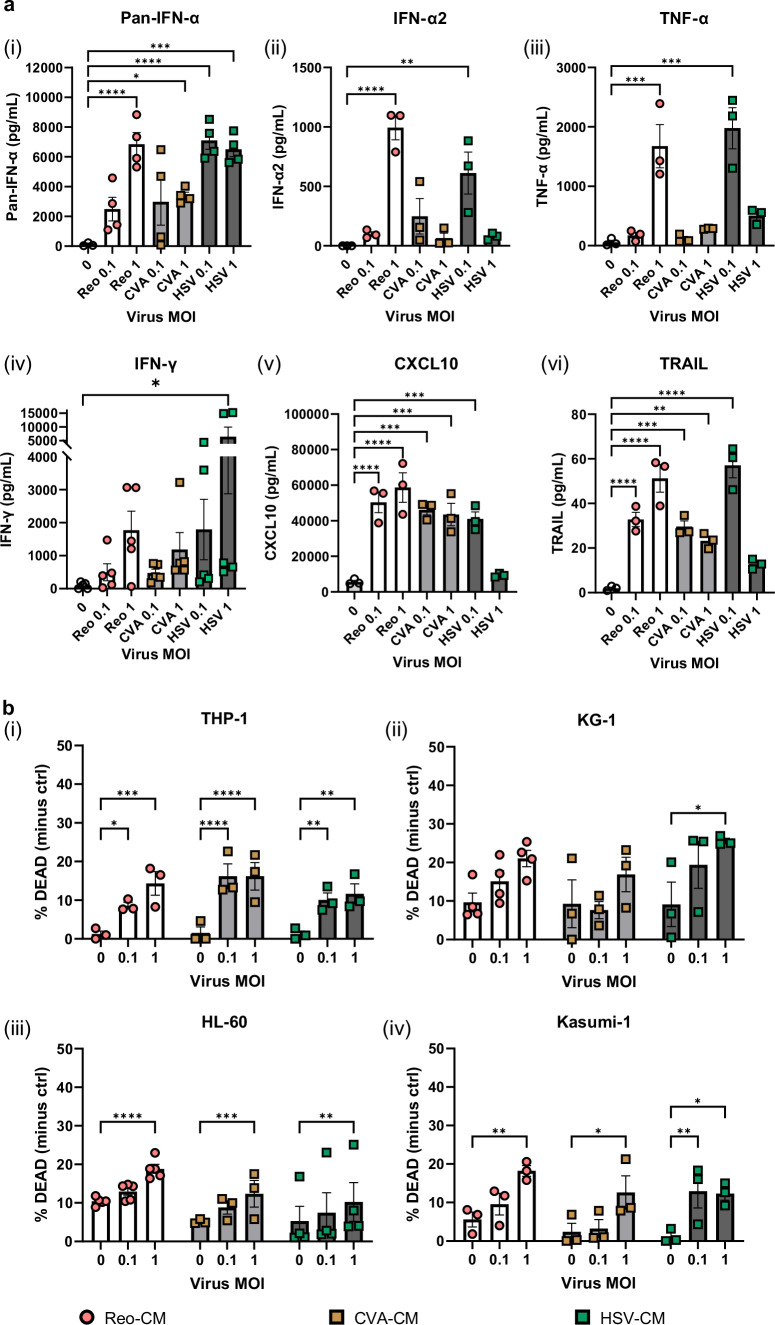
Molecularly distinct OV induce similar cytokine profiles and stimulate AML cell death. Healthy-donor PBMCs were treated with 0, 0.1 and 1 pfu/cell reovirus, CVA21 and HSV-1 for 48 h and cell-free supernatant was collected. **a** Pan-IFN-α, TNF-α and IFN-γ production were measured by ELISA and CXCL10, TRAIL and IFN-α2 were quantified using a Biorad multiplex assay. Bars show the mean ± standard error of the mean (SEM) for *n* ≥ 3 PBMC donors. Statistical significance was determined using a one-way ANOVA. **b** Untreated (0 pfu/cell) or OV-treated (0.1 or 1 pfu/cell) PBMC-CM was added to THP-1, HL-60, KG-1 and Kasumi-1 cells at a 1:1 *v/v* ratio, and cell death was quantified 72 h post-treatment using LIVE/DEAD viability stain. Bars show the mean percentage of dead cells, minus the untreated control (no PBMC-CM) ± SEM (*n* = 3). Statistical significance was determined using a two-way ANOVA and Dunnett’s post-test. *p < 0.05, **p < 0.01, ***p < 0.001, ****p < 0.0001.

OVs can synergise with SMAC/BH3 mimetics to enhance cell death in a cytokine-dependent manner. Primary mediators of this response have included type I IFNs, TNF-α and TRAIL [35–37] therefore we examined whether human PBMCs (±OV treatment) could produce these inflammatory cytokines, alongside other cytokines known to induce extrinsic apoptosis (CXCL10/IFN-γ) (Fig. 1a). Using a range of molecularly distinct OVs (reovirus [double-stranded [ds]RNA], CVA21 [single-stranded [ss]RNA] and HSV-1 [DNA]) we demonstrated that: (i) reovirus and HSV-1 stimulated significant secretion of IFN-α and TNF-α (Fig. 1ai–iii); (ii) all OV-induced significant levels of CXCL10 and TRAIL (Fig. 1av, vi), and (iii) HSV-1 was the only virus to induce significant levels of IFN-γ (Fig. 1aiv). Moreover, none of the OV tested decreased the viability of PBMC, 48 h post-treatment (Supplementary Fig. 2). These data confirmed the inflammatory nature of OV and suggested that OV-treated PBMC may facilitate bystander cytokine-mediated killing of AML. To test this, PBMC-CM (±OV treatment) was harvested, added to AML cell lines at a 1:1 *v/v* ratio, and cell death was quantified (Fig. 1b). Significant cell death was observed for THP-1, HL-60 and Kasumi-1 cells with PBMC-CM generated from all OV tested (Fig. 1bi, iii, iv); by contrast, only HSV-PBMC-CM induced significant killing of KG-1 cells (Fig. 1bii). As expected, based on previous work [27, 28, 42], the cytotoxic effect induced by OV-treated PBMC-CM was low (~10–20%); this correlated with the levels of cell death observed following treatment with combined recombinant cytokine (IFN-α, TNF-α and IFN-γ) which were used at maximum levels observed within OV-treated PBMC-CM (Supplementary Fig. 3). Given the inflammatory and cytotoxic nature of PBMC-CM generated for each OV, we prioritised reovirus for further evaluation based on: (i) its established safety profile in immunosuppressed patients following intravenous delivery [43]; (ii) our previous work reporting its oncolytic and immunostimulatory properties in AML [32]; and (iii) the immunostimulatory properties of UV-inactivated reovirus [41, 44], a potentially safer approach for immunosuppressed patients.

**Fig. 2:**
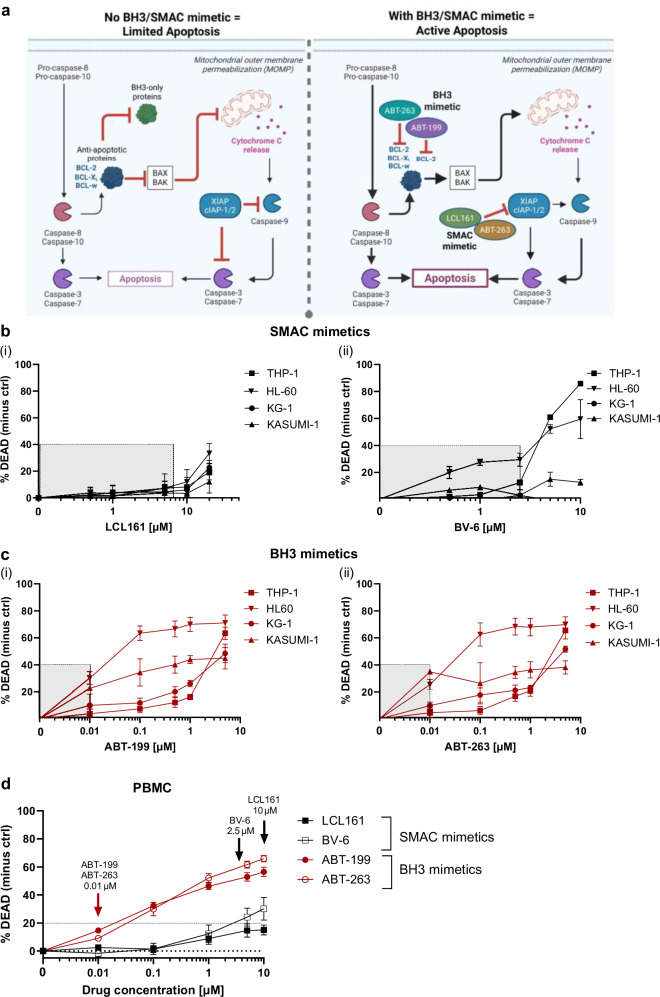
Identify sub-toxic doses of SMAC and BH3 mimetics for combination with OV. **a** Illustration demonstrating (i) the inhibition of apoptosis by anti-apoptotic BCL-2 family members and IAPs (left panel) and (ii) the binding partners of SMAC (LCL161/BV-6) and BH3 (ABT-263/ABT-199) mimetics, which sequester anti-apoptotic proteins and promote apoptosis (right panel). This figure was generated using BioRender.com. THP-1, KG-1, HL-60 and Kasumi-1 cells were treated with increasing doses of **b** SMAC mimetics (LCL161/BV-6) and **c** BH3 mimetics (ABT-199/ABT-263) for 72 h. Cell death was assessed using LIVE/DEAD viability stain. Data shows the mean percentage of cell death for *n* = 3 independent experiments (±SEM), minus the vehicle control. **d** PBMC were treated with SMAC or BH3 mimetics for 72 h, and cell death was measured using LIVE/DEAD viability stain. The data shows the mean cell death (±SEM) for *n* = 3 independent PBMC donors.

To establish sub-toxic doses (<40% death to allow synergistic/additive cytotoxicity to be observed) of SMAC and BH3 mimetics to use in combination with cytokine rich PBMC-CM, AML cell lines were treated with increasing doses of LCL161/BV-6 (SMAC mimetics) or ABT-199/ABT-263 (BH3 mimetics); Fig. 2a illustrates the molecular targets for each drug and their impact on apoptotic signalling. AML cells were relatively resistant to LCL161 (Fig. 2bi); however, >40% cell death was observed in at least one AML cell line following treatment with ≥5 µM BV-6 (Fig. 2bii) or ≥0.1 µM ABT-199/ABT-263 (Fig. 2ci, ii). Cytotoxicity against HD PBMC (essential mediators of OV-induced cytokines) was also determined, and working drug concentrations (0.01 µM ABT-199/ABT-263, 2.5 µM BV-6 and 10 µM LCL161) were selected based on ≤20% PBMC cell death to ensure PBMC viability in the presence of drug (Fig. 2d). Notably, HD CD14+ monocytes were not killed by either SMAC/BH3 mimetics, even at high concentrations (Supplementary Fig. 4).

**Fig. 3:**
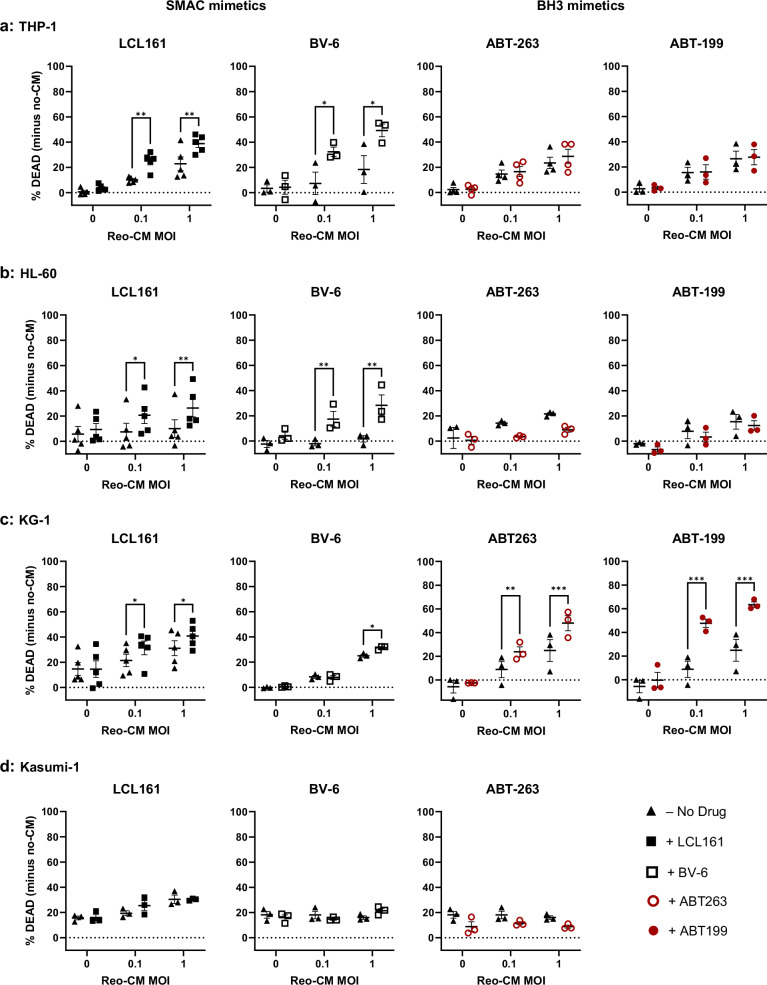
SMAC/BH3 mimetics potentiate reovirus cytokine-mediated killing in AML. **a** THP-1, **b** HL-60, **c** KG-1, and **d** Kasumi-1 cells were treated with 10 µM LCL161, 2.5 µM BV-6, 0.01 µM ABT-199 or 0.01 µM ABT-263 and UV-inactivated PBMC-CM (±0.1 or 1 pfu/cell reovirus; Reo-CM) for 72 h, and cell death was assessed using LIVE/DEAD viability stain. Data shows the mean percentage of dead cells, minus the percentage of dead cells obtained in the absence of PBMC-CM. Data were generated using Reo-CM collected from *n* ≥ 3 individual PBMC donors (±SEM). Statistical significance was performed using two-way ANOVA and Sidak’s post-test comparing the effect of Reo-CM in the presence or absence of the drug. *p < 0.05, **p < 0.01 and ***p < 0.001.

To determine whether SMAC/BH3 mimetics could potentiate reovirus-bystander cytokine-mediated killing, AML cell lines were treated with PBMC-CM (±SMAC/BH3 mimetics) and cell death was quantified. Interestingly, for THP-1 cells, Reo-CM-induced cell death was significantly enhanced in the presence of SMAC mimetics, LCL161 and BV-6, whilst BH3 mimetics had no effect (Fig. 3a); similar results were observed for HL-60 cells (Fig. 3b). By contrast, the opposite effect was observed for KG-1 cells and BH3 mimetics were more effective at increasing Reo-CM-induced cell death (Fig. 3c); this effect was more pronounced for ABT-199, which targets BCL-2. Unfortunately, none of the compounds tested were effective in Kasumi-1 cells (Fig. 3d). Using THP-1 cells we also confirmed that LCL161 significantly enhanced cell death in combination with HSV-1-CM or CVA21-CM, whilst ABT-263 was ineffective (Supplementary Fig. 5). These data: (i) confirmed that apoptotic modulators could be used to potentiate OV-induced bystander cytokine-mediated killing, and (ii) suggested that different combination partners were required for distinct AML subtypes.

**Fig. 4:**
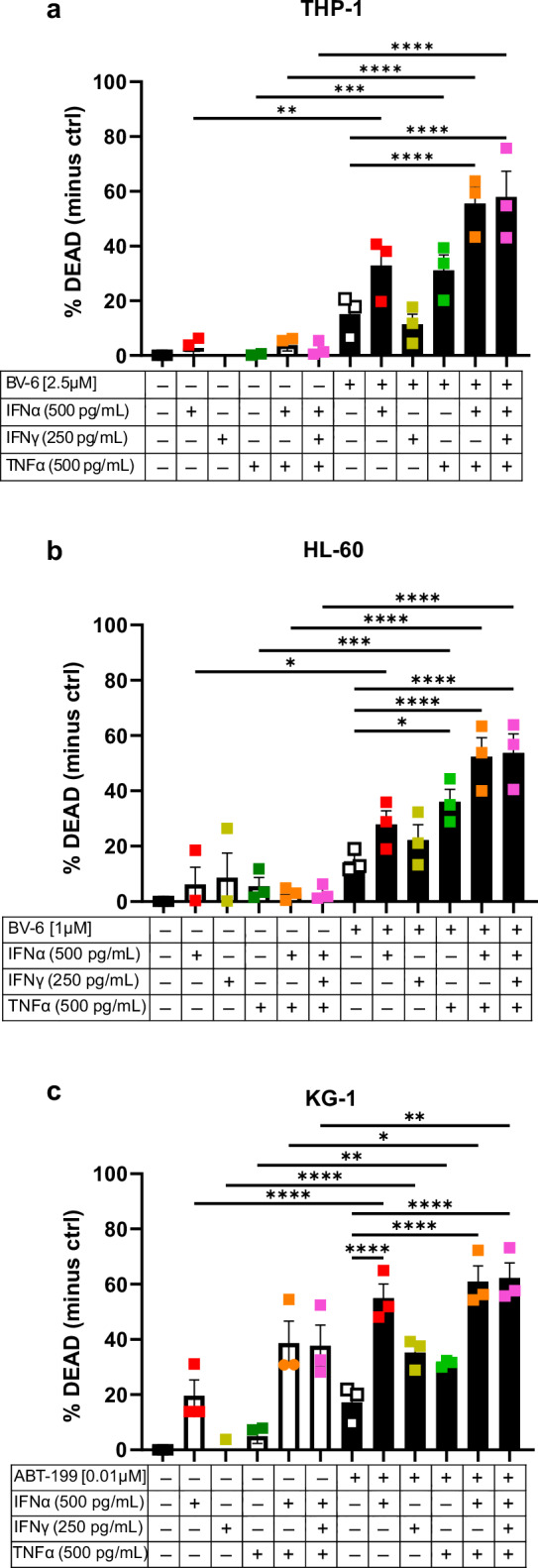
AML cell death is mediated by multiple reovirus-induced cytokines. AML cell lines were treated with 500 pg/mL IFN-α, 250 pg/mL IFN-γ and 500 pg/mL TNF-α alone, or combinations thereof, including dual IFN-α/ TNF-α (500 pg/mL each) or triple IFN-α/IFN-γ/TNF-α (500 pg/mL IFN-α/TNF-α plus 250 pg/mL IFN-γ), ± optimal SMAC/BH3 mimetics for 72 h: **a** THP-1 cells were treated with 2.5 µM BV-6; **b** HL-60 cells were treated with 2.5 µM BV-6; and **c** KG-1 cells were treated with 0.01 µM ABT-199. Cell death was measured by LIVE/DEAD viability stain, and the mean percentage of dead cells (minus the vehicle control) is shown for *n* = 3 experiments (±SEM). Statistical significance was performed using one-way ANOVA and Tukey’s post-test; *p < 0.05, **P < 0.01, ***p < 0.001, ****p < 0.0001.

OVs stimulate the production of numerous cytokines (Fig. 1); therefore, we sought to determine which reovirus-induced inflammatory mediators were important for AML cell death when combined with the optimal SMAC/BH3 mimetics (identified in Fig. 3). We initially focused on IFN-α, TNF-α and IFN-γ as they are upregulated following OV treatment [27, 28] and can synergise with SMAC mimetics [35, 36]. Figure 4a shows that THP-1 cells were not sensitive to IFN-α, TNF-α or IFN-γ killing, alone or in combination; however, in the presence of BV-6, a significant increase in THP-1 cell death was observed with IFN-α and/or TNF-α, but not IFN-γ. Moreover, BV-6 combined with dual IFN-α/TNF-α treatment was more effective than BV-6 plus IFN-α (*p* = 0.04) or TNF-α (*p* = 0.02), with no further benefit of IFN-γ; similar data was obtained using LCL161 (Supplementary Fig. 6). Like THP-1 cells, HL-60 cells were not sensitive to cytokine-mediated killing in the absence of BV-6; however, BV-6 enhanced killing in the presence of TNF-α and dual IFN-α/TNF-α treatment (Fig. 4b). By contrast, KG-1 cells were more susceptible to cytokine-mediated killing in the absence of drug, with significant cell death observed with dual IFN-α/TNF-α treatment (*p* < 0.0001). Nonetheless, ABT-199 enhanced cell death in the presence of both IFN-α and dual IFN-α/TNF-α treatment, with no significant effects observed for TNF-α or IFN-γ (Fig. 4c). These data suggest a role for both TNF-α and/or IFN-α in mediating AML cell death in combination with SMAC/BH3 mimetics; however, this does not negate the involvement of other cytokines not evaluated in this study.

Our previous studies have shown that reovirus infection of AML cell lines (THP-1/KG-1) results in IFN-α secretion [32]; therefore, we hypothesised that combination with SMAC/BH3 mimetics would enhance cell death, even in the absence of PBMC-CM. Reovirus-mediated killing was confirmed in all cell lines (Supplementary Fig. 7). Moreover, a significant increase in cell death was observed in combination with BV-6 for THP-1/HL-60 cells (Supplementary Fig. 7a, b) and ABT-199 for KG-1 cells (Supplementary Fig. 7c). Notably, this was particularly pronounced for THP-1 and KG-1 cells, which produce ~500 pg/mL IFN-α following reovirus infection [32].

**Fig. 5:**
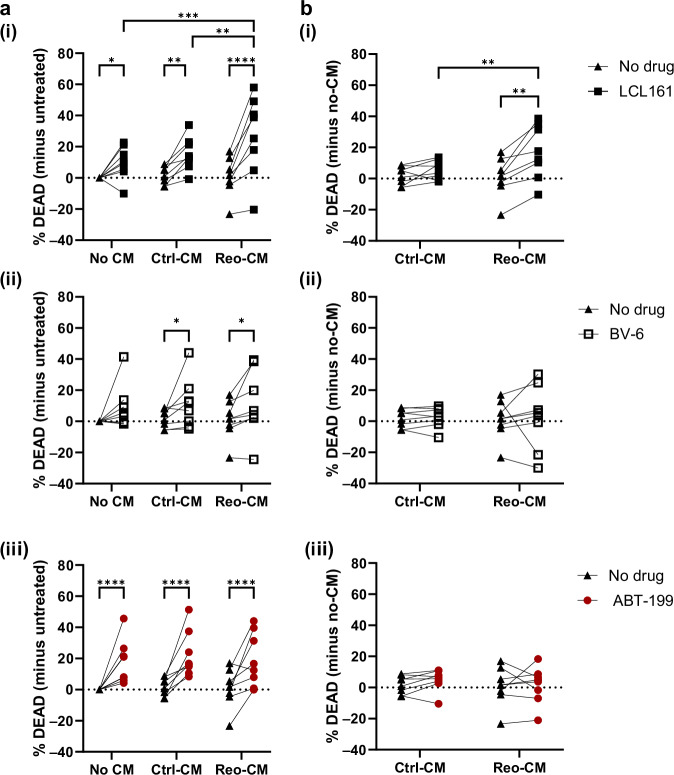
LCL161 enhances reovirus cytokine-mediated killing in AML patient samples. PBMC were isolated from AML patient samples and treated with 1 µM LCL161, 0.1 µM BV-6 or 0.001 µM ABT-199 in the presence or absence of HD PBMC-CM (±reovirus treatment) for 48 h (*n* = 8); cell death was assessed using LIVE/DEAD viability stain. Note, the concentration of the drug used on AML cell lines was too toxic to observe combinatorial effects on patient samples. **a** The percentage of dead cells (minus untreated vehicle control) after treatment with drug and/or HD PBMC-CM (±reovirus treatment) is shown. **b** The percentage of dead cells (minus values obtained in the absence of PBMC-CM) following treatment with drug and HD PBMC-CM (±reovirus treatment). Statistical significance was performed using a two-way ANOVA with Sidak’s post-test. *p < 0.05, **p < 0.01, ***p < 0.001 and ****p < 0.0001

Next, the ability of SMAC/BH3 mimetics to enhance reovirus cytokine-mediated killing was examined using primary AML samples (Supplementary Table 4) and HD PBMC-CM, ±reovirus treatment. To normalise for differences in cell viability across each sample, we initially subtracted the percentage of dead cells observed in untreated controls (Fig. 5ai–iii). As expected, all drugs induced significant AML cell death, although the cytotoxic effect of ABT-199 was more pronounced, with a mean increase of 17.6% versus 9.8% and 8.7% for LCL161 and BV-6, respectively. Unfortunately, the cytotoxic effect of Reo-CM versus Ctrl-CM (in the absence of drug) was not significant, and enhanced killing in the presence of Reo-CM was only observed with LCL161 (Fig. 5ai). To confirm this, and remove the effect of the drug alone, data were normalised to remove the percentage of dead cells observed in the absence of PBMC-CM (Fig. 5b). These data demonstrate a significant increase in AML cell death in the presence of Reo-CM and LCL161 (Fig. 5bi) but not BV-6 (Fig. 5bii) or ATB-199 (Fig. 5biii). These data suggested that LCL161 was more effective than BV-6, which was unexpected given the data presented in Fig. 3.

**Fig. 6:**
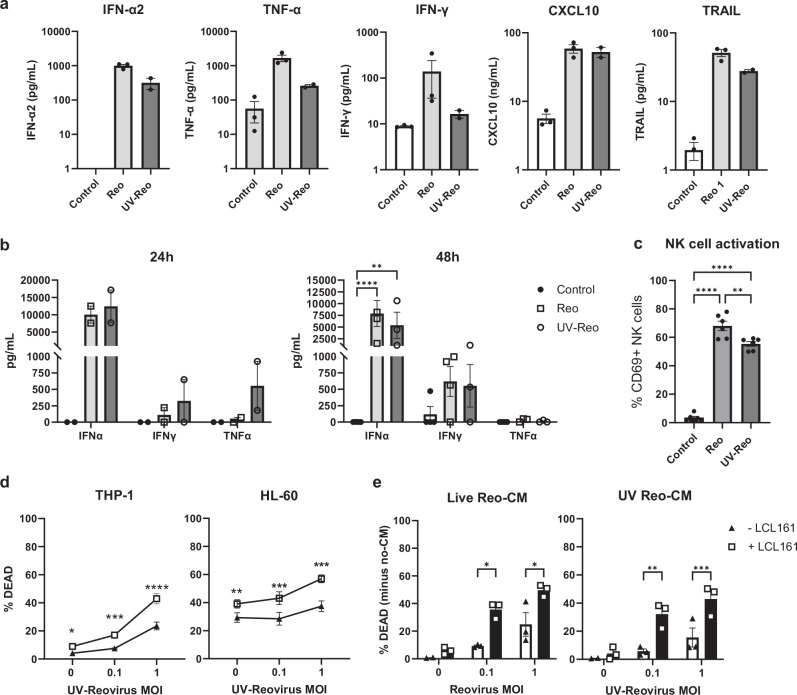
UV-inactivated reovirus can induce inflammation and kill AML cells in the presence of apoptotic modulators. **a** HD PBMC were treated for 48 h with 1 pfu/PBMC live (Reo, *n* = 3—reproduced from Fig. 1 for comparison) or UV-inactivated Reovirus (UV-Reo, *n* = 2), and cytokines were measured using a Biorad multiplex immunoassay; bar charts show concentrations in pg/mL, ±SEM. **b** PBMC were isolated from AML patient samples and treated with 1 pfu/cell Live-Reo or UV-Reo for 24 h (*n* = 2) for 48 h (*n* ≥ 3), and pan-IFN-α, TNF-α and IFN-γ production were measured by ELISA. **c** PBMCs isolated from an AML patient were treated with 1 pfu/PBMC Live-Reo or UV-Reo for 48 h, and NK cell activation was measured by flow cytometry; NK cells were identified as CD3^−^CD56^+^ cells, and the percentage of NK cells expressing CD69 was determined. Error bars indicate mean ± SEM for *n* = 6 patient samples. **d** THP-1/HL-60 cells were treated with 0, 0.1 or 1 pfu/cell UV-Reo for 24 h followed 10 µM LCL161 for a further 48 h. Cell death was assessed using LIVE/DEAD viability stain, and the mean percentage of dead cells is shown for *n* ≥ 3 experiments, ±SEM. **e** PBMCs were treated with Live-Reo or UV-Reo for 48 h, and the supernatant was collected. THP-1 cells were treated with PBMC-CM (Live-Reo-CM or UV-Reo-CM) in the presence of 10 µM LCL161 for 72 h, and cell death was assessed using LIVE/DEAD viability stain. The mean percentage of dead cells after treatment with Live-Reo-CM or UV-Reo-CM is shown for *n* = 3 independent experiments, ±SEM. Statistical significance was performed using a one or two-way ANOVA with Sidak’s or Tukey’s post-test, as appropriate. *p < 0.05, **p < 0.01, ***p < 0.001 and ****p < 0.0001.

The absence of differentiated myeloid cells, which coordinate reovirus anti-tumour and anti-viral immunity [44], could limit the clinical utility of reovirus due to the concerns relating to uncontrolled viral replication. Therefore, we investigated whether UV-inactivated reovirus (UV-Reo) would be effective in combination with LCL161. Figure 6a shows inflammatory cytokine production from HD PBMC after treatment with Live-Reo (replication-competent) or UV-inactivated (replication-defective) reovirus. Importantly, (i) all cytokines were produced although, except for CXCL10, levels were lower for UV-Reo; (ii) IFN-α was produced from AML patient samples, with no significant difference between live- and UV-Reo (Fig. 6b), and (iii) increased CD69 expression on patient NK cells was observed, in agreement with the role for IFN-α for NK cell activation (Fig. 6c). Critically LCL161 enhanced cell death following direct treatment of THP-1/HL-60 cells with UV-Reo (Fig. 6d) and increased THP-1 cell death in combination with PBMC-CM generated using Live- or UV-Reo. These data confirm that the effect of LCL161 on bystander cytokine-mediated killing is maintained in the absence of reovirus replication.

## Discussion

Pro-inflammatory cytokines induced by OV-treated PBMC-CM had some, albeit low, anti-AML activity; however, this was enhanced with SMAC (LCL161/BV-6) and/or BH3 (ABT-199) mimetics. Accordingly, our previous work demonstrated bystander cytokine-mediated killing of AML and multiple myeloma using CVA21-stimulated and Reo-stimulated PBMC-CM [27, 28]. IFN-α and TNF-α (OV-induced cytokines) have known anti-AML activity and were identified herein as cytotoxic mediators when used in combination with SMAC/BH3 mimetics [23, 45]. However, other OV-induced cytokines might also play a role since synergistic effects in other cancers have been associated with TRAIL, IL-1A and IL-8 [37], as well as IFN-β/TNF-α [36]. In agreement with previous literature [36], autocrine-dependent killing was observed following reovirus infection and was increased with SMAC/BH3 mimetics. This suggests a possible role for reovirus-induced IFN-α [32], which has been detected in patient serum following intravenous reovirus administration [46].

Healthy monocytes were resistant to the SMAC/BH3 mimetics, and limited PBMC toxicity was observed for SMAC mimetics. By contrast, PBMC were more susceptible to BH3 mimetics, reflecting the sensitivity of lymphocytes [47]. In agreement with our data, LCL161 was safe/well-tolerated in clinical trials with only 4–6% of patients developing thrombocytopenia and anaemia [21]. Common adverse events for ABT-199 include diarrhoea, vomiting and neutropenia [48]. Interestingly, a recent report investigating the efficacy of the SMAC mimetic Xevinapant in combination with chemoradiotherapy reported worse overall survival and an unfavourable safety profile in squamous cell carcinoma patients, demonstrating the requirement for clinical evaluation [49]. Importantly, the combination with OV, which are safe/well-tolerated, could allow reduced drug dosing to increase the safety profile of these agents. Moreover, the therapeutic efficacy and approval of Venetoclax (an alternative class of apoptotic modulators) for the treatment of AML is encouraging if we can identify molecular markers of response and stratify patients accordingly.

OV have been well tolerated in multiple myeloma and cutaneous T-cell lymphoma patients, and immune activation and/or tumour regression have been reported [43, 50–52]. However, uncontrolled reovirus replication in heavily diseased patients (with limited immune function) could reduce the clinical utility of replication-competent reovirus. Therefore, a personalised treatment strategy could be considered whereby replication-competent reovirus could be employed in combination with SMAC/BH3 mimetics during periods of relative immune competence, for example, after haematological recovery, as a minimal-residual disease-directed strategy. By contrast, SMAC/BH3 mimetics could be used in combination with UV-inactivated reovirus when disease burden is high (and immune function compromised), potentially alongside current therapies. Critically, UV-Reo can mature DC [53], activate NK cells [44] and prime tumour-specific cytotoxic T lymphocytes [41], inducing anti-tumour immune responses beyond those observed with OV-induced cytokines. Accordingly, we have confirmed that: (i) both live- and UV-Reo can activate NK cells from HD PBMC [44] and AML patients (Fig. 6c); (ii) reovirus-activated NK cells can degranulate against AML cells [32]; and (iii) AML cells are susceptible to OV-activated NK cell killing [27]. Pivotally, SMAC mimetic treatment did not abrogate NK cell killing of AML (Supplementary Fig. 8), although, in contrast to published work, enhanced NK cell killing was not observed [54, 55]. Moreover, using CVA21, we have previously shown that OV can prime AML-specific T cells against known TAA, suggesting a potential role of OV-induced T cell-mediated immunity. Importantly, to more effectively harness cell-mediated anti-tumour immune response, it may be necessary to administer this treatment over multiple cycles, where early efficacy relies on autocrine/paracrine cytokine release and enhanced apoptosis in the presence of SMAC/BH3 mimetics. By contrast, when disease burden is low and immune composition is restored, additional cycles of therapy could enhance cytokine-mediated killing, whilst simultaneously activating NK cell-mediated killing or priming AML-specific T cells.

Whilst we have not evaluated the effect of SMAC/BH3 mimetics on AML-specific T cells in vitro or in vivo, a role for adaptive anti-tumour immunity has been reported. Specifically, SM83 prolonged the survival of ovarian cancer-bearing mice and protected against tumour rechallenge, reflecting a T cell-mediated immune response [56]. LCL161 (in combination with VSVΔM51) also reversed CD8+ T cell exhaustion and reduced breast cancer tumour burden in a T cell-dependent manner [57], and synergises with alternative immunotherapies (e.g. immune checkpoint inhibitors) in a TNF-a/IFN-a dependent manner [58]; this alternative combination approach warrants investigation in future AML-focused studies. Unfortunately, in vivo validation was beyond the scope and funding of this project; however, future in vivo studies would provide further mechanistic insight relating to the role of T cells and efficacy in combination with other forms of immunotherapy.

Interestingly, upon assessment of AML patient samples (Supplementary Table 4), LCL161 was more effective at potentiating Reo-CM cytotoxicity. The benefit of LCL161 over BV-6 remains unclear; however, their distinct structural properties, bivalent versus monovalent, are plausible. Unfortunately, ABT-199 only potentiated Reo-CM killing in 1/9 samples; however, it is possible that an ABT-199 ‘responding’ group could be identified if the sample size was increased, given the diversity of disease phenotypes encompassed by AML. By contrast, LCL161 enhanced the cytotoxic effect of Reo-CM in 6/9 patients. Unfortunately, it is difficult to predict genetic markers of susceptibility with the sample number available; however, preliminary findings suggest a potential role for *FLT-3* mutations. Accordingly, synergistic responses with extrinsic apoptotic stimuli (TRAIL/CD95L) and BV-6 have been reported in AML patients harbouring *FLT-3-ITD* mutations [59]. Regarding ABT-199/venetoclax, *IDH2*- and *NPM1*-mutations have been associated with efficacy [60]. However, a recent case report described the efficacy of azacytidine plus venetoclax in a 19-year-old patient with *WT-1*-mutated AML, suggesting a possible role for venetoclax in young patients with *WT-1* mutations, in line with our data [61]. Importantly, both the cell line and AML patient data suggest that different apoptotic modulators would be required for different patients to enhance cytokine-mediated killing following OV treatment. Future studies should increase the AML cell line panel and expand the patient cohort to identify cellular determinants of response to both OV and SMAC/BH3 mimetics. Once identified, AML patients could be screened to identify optimal combination partners, thus offering a more personalised/stratified treatment approach for patients.

The outcome for AML patients remains dismal, and novel and effective therapies are urgently needed. Here, we highlight the therapeutic potential of oncolytic reovirus (or other OV) in combination with apoptotic modulators as a potential novel treatment. This strategy warrants further investigation, alone or in combination with existing therapies.

## Supplementary information

Supplementary Information
