# Supplementary material for: Apoptotic modulators enhance oncolytic virus-induced cytokine killing in acute myeloid leukaemia (AML)
Source: Br J Cancer. 2026 Apr 10;135(1):152–62. doi: 10.1038/s41416-026-03417-x (PMC13269698; doi:10.1038/s41416-026-03417-x)
Supplement: Supplementary file 1 — Supplementary Information [file 41416_2026_3417_MOESM1_ESM.pdf]

SUPPLEMENTARY METHODS

CD14 selection: CD14 isolation was performed using CD14 MACS microbeads (Miltenyi Biotec Ltd.) according to the manufacturer's instructions. In brief, healthy donor PBMC were washed in MACS buffer (PBS; 1% FCS; 2 mM EDTA), resuspended in 80 µL of MACS buffer/20 µL per 1x10^7 cells and incubated for 15 minutes at 4°C. Cells were washed in an excess volume of MACS solution, re-suspended in 500 µL of MACS buffer per 1x10^8 cells and labelled cells were isolated using MACS® LS separation columns. After washing, CD14+ cells were removed from the column using MACS buffer and cells were resuspended in RPMI-1640 supplemented with 10% FCS at 1x10^6 cells/mL.

^{51}Chromium release assay to assess NK cell cytotoxicity: 1x10^6 target cells were harvested and labelled for one hour at 37°C with 100 µCi ^{51}Cr (PerkinElmer). After labelling, the target cells were washed (x3) in PBS and resuspended at 5x10^4 cells/mL in RPMI-1640 supplemented with 10% FCS. The effector PBMC (± reovirus treatment) were harvested, resuspended at 5x10^6 cells/mL in RPMI-1640 plus 10% FCS and 200µL of cells were added in triplicate to 96-well round-bottom plate. To generate different effector:target (E:T) ratios, beginning at 100:1, halving serial dilutions of effector PBMC were made using 100µL RPMI-1640. 5x10^3 ^{51}Cr labelled target cells were added to each well and co-cultured for 4 hours (37°C). Spontaneous release was measured using separate plate where 100µL ^{51}Cr-labelled target cells were placed in media alone. To quantify maximum release, 100µL ^{51}Cr-labelled cells were placed in media containing 1 % Triton X (Sigma-Aldrich). After the 4 (37°C), cells were pelleted at 400g for 5 minutes and 50 µL of the supernatant was transferred to a Lumaplate (Perkin Elmer). Plates were dried overnight ^{51}Cr levels were quantified using using a Microbeta2 scintillation counter (PerkinElmer). The percent of target cell lysis was determined using the following formula:

$$\% \, ^{51}\text{Cr Release} = \frac{\text{sample (counts per minute; cpm) - spontaneous release (cpm)}}{\text{maximum release (cpm) - spontaneous release (cpm)}} \times 100$$

SUPPLEMENTARY TABLES

Target	Fluorochrome	Volume added	Origin	clone	Supplier
IgG control	PE	5 µL	mouse	SK7	Biolegend
CD3	PerCP	5 µL	mouse	W264/56	Biolegend
CD56	eFluor450	2 µL	mouse	TULY56	Miltenyi Biotec
CD69	PE	5 µL	mouse	FN50	Biolegend
CD45	FITC	2 µL	mouse	REA293	Miltenyi Biotec

Supplementary Table 1: Flow cytometry antibodies. PE: phycoerythrin, PerCP: peridinin chlorophyll protein complex, FITC: fluorescein isothiocyanate.

Target molecule	Species of origin	Clone	Role	Dilution	Dilution buffer
IFN-α	Mouse	MT1/3/5	Capture	1:250	PBS
IFN-α	Mouse	MT2/4/6	Detection	1:1000	10% FCS in PBS
IFN-γ	Mouse	NIB42	Capture	1:250	100nM $NaHCO_3$ in ddH2O
IFN-γ	Mouse	4S.B3	Detection	1:500	10% FCS in PBS
TNF-α	Mouse	Mab1	Capture	1:500	100nM $NaHCO_3$ in ddH2O
TNF-α	Mouse	68B3C5	Detection	1:1000	10% FCS in PBS

Supplementary Table 2: Matched-paired ELISA Antibodies. IFN-a, MabTech; IFN-g and TNF-a, BD Biosciences

Cytokine	Species	Top standard concentration	Bottom standard concentration	Manufacturer
IFN-α	Human	10000 pg/mL	156.25 pg/mL	BD Biosciences
IFN-γ	Human	4000 pg/mL	62.5 pg/mL	BD Biosciences
TNF-α	Human	2000 pg/mL	31.25 pg/mL	Biosource

Supplementary Table 3: Recombinant standards for ELISA.

Age	Diagnosis	Sample	% neoplastic cells	Significant Mutations	% increase in cell death with LCL161	% increase in cell death with ABT199
43	AML with mutated NPM1	Diagnosis	74	FLT3, IDH1, DNMT3A and NPM1	35.27	6.53
51	AML with MDS-related changes	Diagnosis	28	Unknown.	17.6	-1.64
72	AML arising from transformation of MDS/MPN	Diagnosis. Previously had MPN.	85	FLT3, IDH2 and SRSF2	12.39	3.76
49	AML	Diagnosis	>30	FLT3	31.44	4.97
20	AML with biallelic mutations of CEBPA	Diagnosis	61	CEBPA and WT-1	10.39	18.32
60	AML with mutated NPM1	Diagnosis	5.50	FLT3, DNMT3A, NPM1	0.75	-6.96
48	AML with biallelic mutations of CEBPA	Diagnosis	65	CEBPA, GATA2- and KRAS.	-10.35	-21.05
82	AML NOS	Diagnosis	42	FLT3, NRAS, TET2 and WT1.	38.57	8.66

Supplementary Table 4: AML patient characteristics and percent increase in Reo-CM cell death with LCL161 or ABT-199 per patient.

SUPPLEMENTARY FIGURES

UV-inactivated Reo-CM **Non-UV-inactivated Reo-CM**

-1

-2

-3

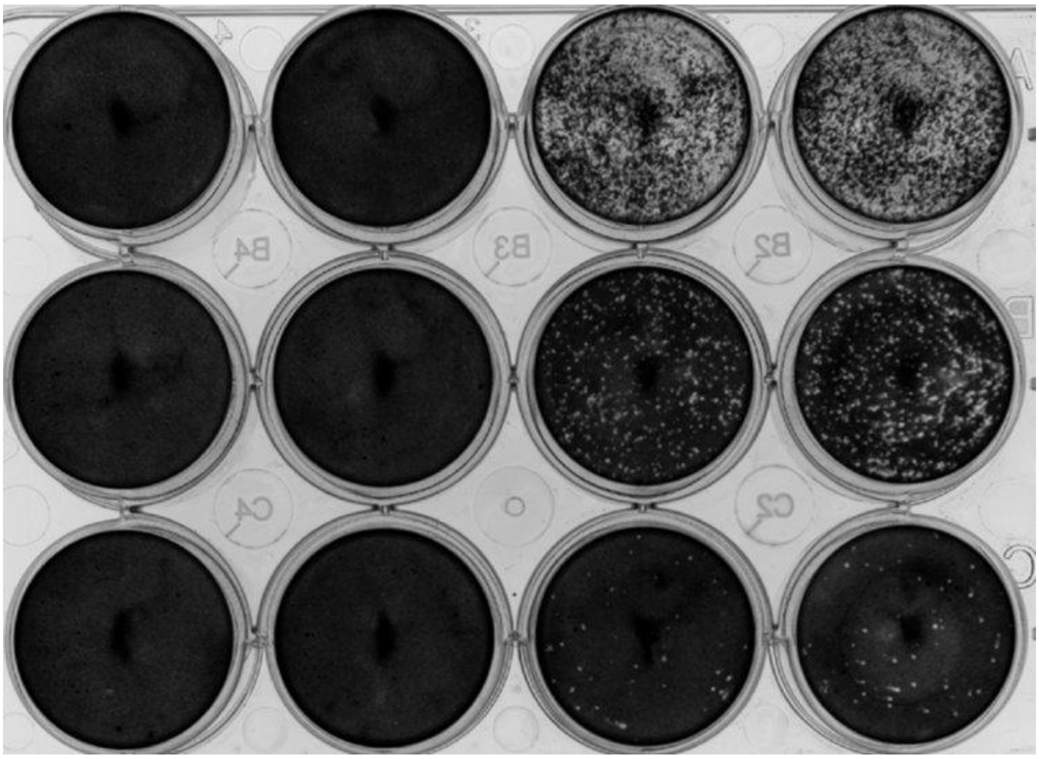

UV-inactivated HSV-CM **Non-UV-inactivated HSV-CM**

-1

-2

-3

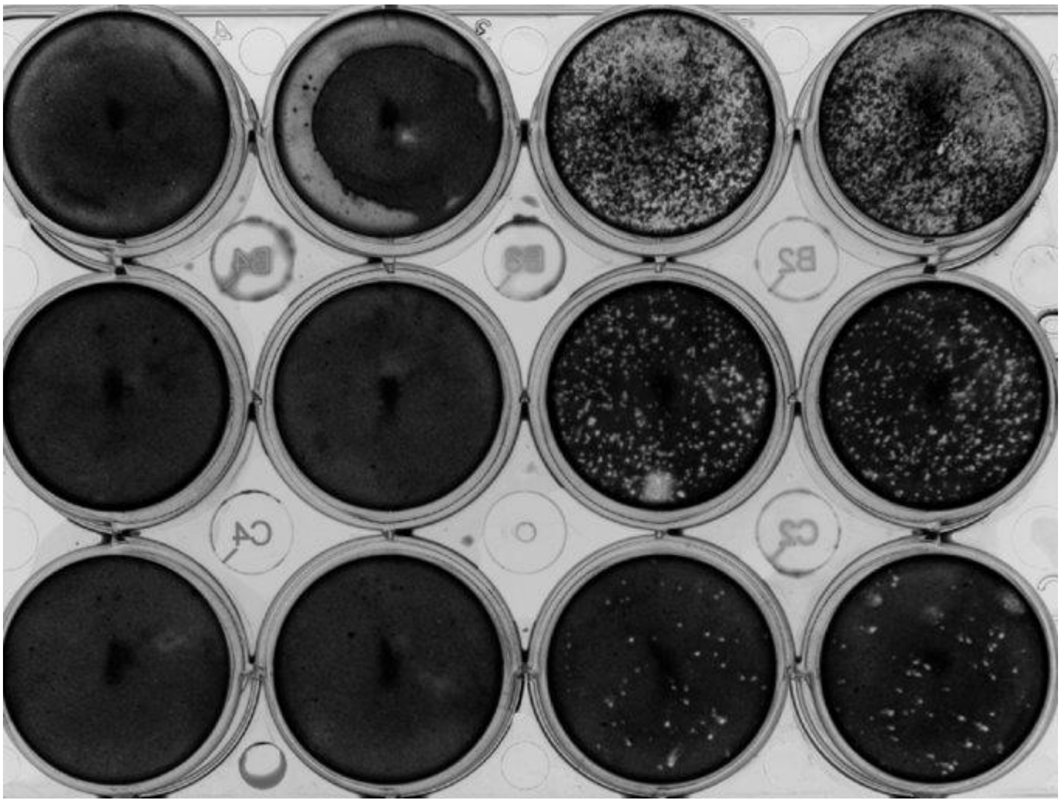

Supplementary Figure 1: UV-inactivation of reovirus and HSV-1. PBMC donors (n=3) were treated with 1PFU/cell reovirus or HSV-1 for 48hr and cell-free supernatant was collected and stored at -80°C. Cells for plaque assays (L929 for Reovirus and Vero for HSV-1) were seeded and left to adhere overnight. OV-treated PBMC-CM were thawed and UV-inactivated for 2 mins or left at room temperature for 2 mins, as a control. UV-inactivated and non-UV-inactivated samples were serially diluted 10-fold (-1) up to 1000-fold (-3) in serum-free media and standard plaque assays were performed for each virus. After 72 hr, cells were washed with PBS, fixed in 1% PFA and stained with 1% methylene blue before being washed with water and images taken. Representative images for each virus (reovirus and HSV-1) are shown for n=3 independent PBMC donors.

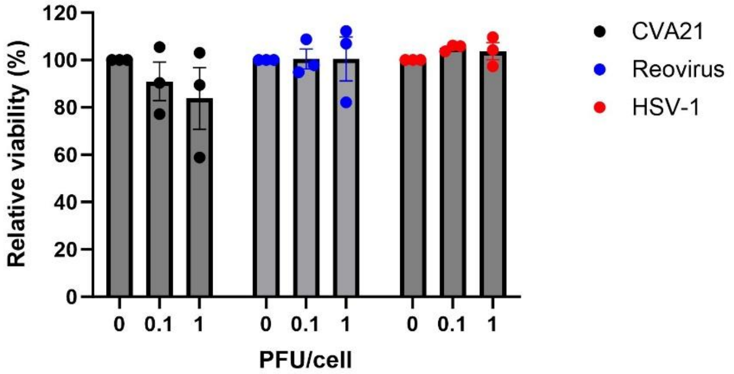

Supplementary Figure 2: OV treatment does not decrease PBMC viability. PBMC donors (n=3) were treated with 0.1 or 1PFU/cell CVA21, reovirus or HSV-1 for 48 hr and cell viability was quantified using LIVE/DEAD flow cytometry. Data shows the mean percentage of viable PBMC, relative to untreated PBMC, for each virus (± SEM). No statistically significant differences were observed.

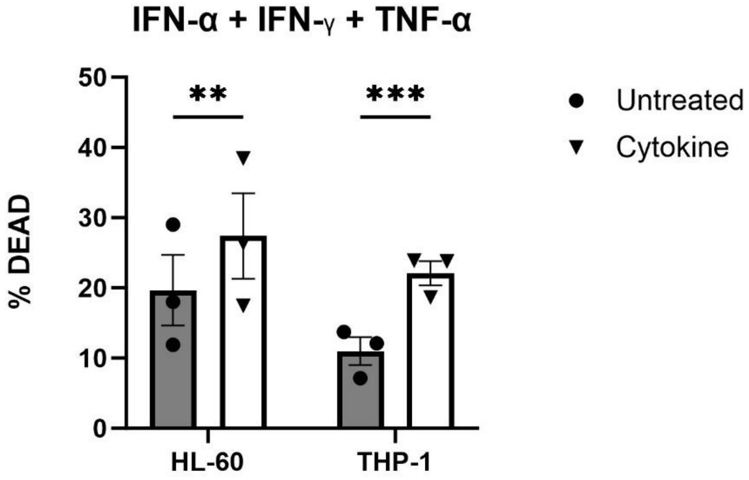

Supplementary Figure 3: Combined recombinant cytokines induce AML cell death. HL-60 and THP-1 cells were treated with 2000pg/mL IFN-α/TNF-α and 1000pg/mL IFN-γ for 72 hr and cell death was measured using a LIVE/DEAD viability stain. The mean percentage of dead cells is shown for n=3 experiments (±SEM). Statistical significance was performed using two-way ANOVA and Sidak's post-test; **=P<0.01, ***=p<0.001.

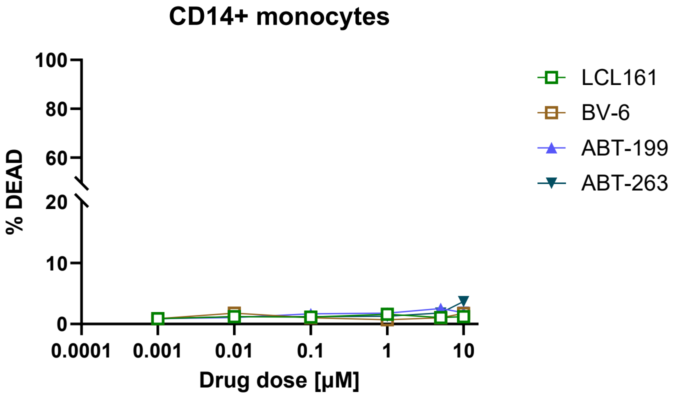

Supplementary Figure 4: Cytotoxicity of SMAC/BH3 mimetics on healthy myeloid cells. PBMC were prepared from healthy donor blood and CD14+ monocytes were isolated using MACS selection (see supplementary methods for CD14 isolation protocol). CD14+ cells were treated with SMAC or BH3 mimetics for 72 hr and cell death was measured using LIVE/DEAD flow cytometry. The data shows the mean cell death (± SEM) for n=3 independent PBMC donors.

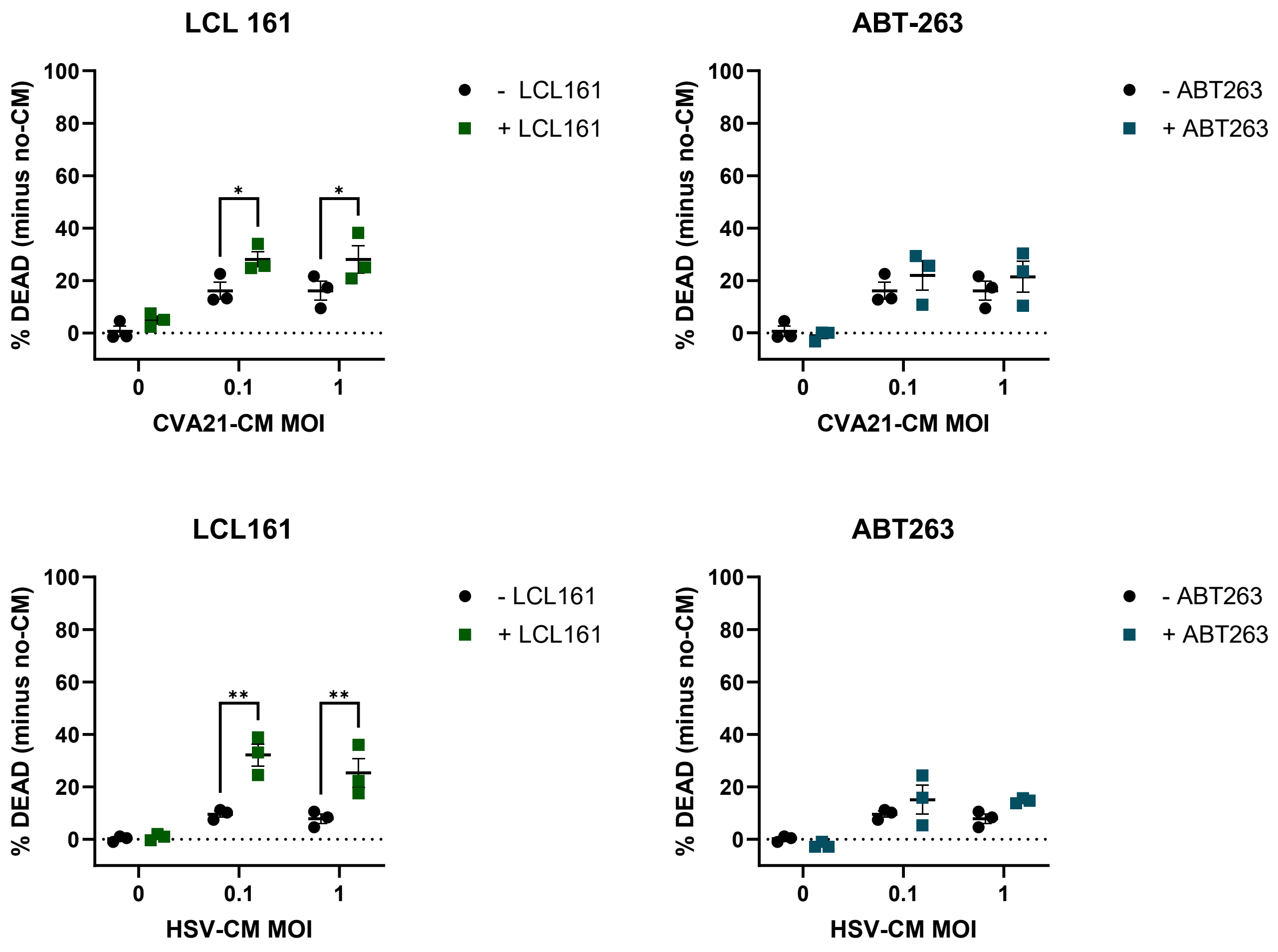

Supplementary Figure 5: SMAC/BH3 mimetics potentiate reovirus-induced cytokine killing in AML. THP-1 cells were treated with 10 µM LCL161 or 0.01 µM ABT263 and UV-inactivated PBMC-CM (± HSV-1 or CVA21 treatment at 0.1 or 1pfu/cell, HSV-CM and CVA21-CM, respectively) for 72 hr and cell death was assessed using LIVE/DEAD flow cytometry. Data shown are the mean percentage (± SEM) of dead cells following treatment with PBMC-CM collected from n=3 PBMC donors, minus the percentage of dead cells obtained in the absence of PBMC-CM. Statistical significance was performed using two-way ANOVA and Sidak post-test comparing the effect of OV-CM in the presence (coloured) or absence (black) of drug. *=p<0.05, **=p<0.01 and ***=p<0.001.

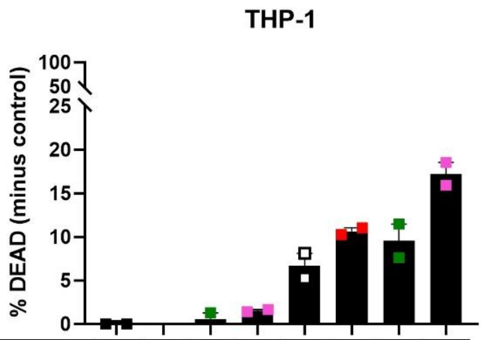

LCL161 [20µM]	-	-	-	-	+	+	+	+
IFNα (1000 pg/mL)	-	+	-	+	-	+	-	+
TNFα (1100 pg/mL)	-	-	+	+	-	-	+	+

Figure 6: AML cell death is mediated by reovirus-induced cytokines. THP-1 cells were treated with 1000pg/mL IFN-α or TNF-α alone or in combination, ± LCL161 for 72 hr. Cell death was measured by LIVE/DEAD viability stain and the mean percentage of dead cells (minus the vehicle control) is shown for n=2 experiments (±SEM). One-way ANOVA and Tukey's post-test confirmed a significant increase in cell death between LCL161 alone and LCL161 plus dual IFN-α/TNF-α ($p=0.0003$).

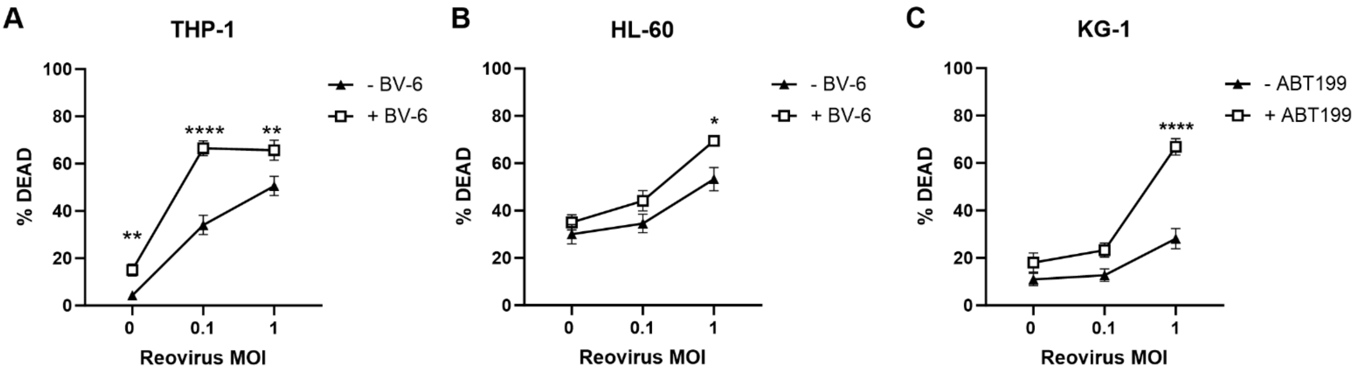

Supplementary Figure 7: Apoptotic modulators enhance direct reovirus killing in the absence of PBMC. A: THP-1, **B**: HL-60 or **C**: KG-1 cells were treated with 0, 0.1 or 1 pfu/cell reovirus for 24 hr followed 2.5 µM BV-6 (**A/B**) or 0.01 µM ABT-199 (**C**) for a further 48 hr. Cell death was assessed using LIVE/DEAD viability stain and the mean percentage of dead cells is shown for n≥3 experiments, ±SEM. Statistical significance in the presence or absence of drug was performed using a two-way ANOVA with Sidak's post-test. *=$p<0.05$, **=$p<0.01$, ***=$p<0.001$ and ****=$p<0.0001$.

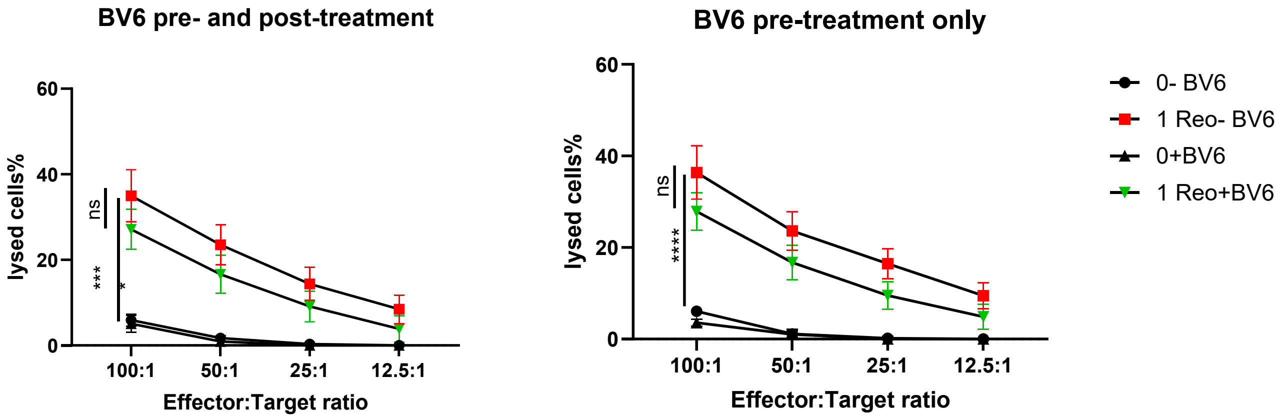

Supplementary Figure 8: NK cell-mediated killing of THP-1 is not abrogated by BV-6.HD-PBMC were treated with 0 or 1pfu/cell Reo for 48 hr. THP-1 cells were treated with 2.5 µM BV-6 for 24 hr and labelled with ^{51}Cr for 1 hour prior to washing. ^{51}Cr-labelled THP-1 cells (±BV-6 treatment) were: **A**: co-cultured with PBMC in the absence of BV-6 or **B**: maintained in BV-6 upon co-culture with PBMC. After 5 hr, THP-1 cell lysis was evaluated using a ^{51}chromium release assay at various effector:target ratios. Error bars indicate mean ± SEM for 6 PBMC donors. Statistical significance was performed using a two-way ANOVA. ns indicates that there was no significant difference in the percentage of lysed cells following treatment with 1 pfu/cell Reo in the presence (green) or absence (red) of BV-6.
